# Correlation between hepatic human males absent on the first (hMOF) and viral persistence in chronic hepatitis B patients

**DOI:** 10.1186/s13578-018-0215-5

**Published:** 2018-02-20

**Authors:** Liwen Chen, Chuanwu Zhu, Fengdi Li, Yun Wang, Rebecca Bao, Zhujun Cao, Xiaogang Xiang, Lei Yan, Lanyi Lin, Gangde Zhao, Qing Xie, Shisan Bao, Hui Wang

**Affiliations:** 1 Department of Infectious Diseases, Ruijin Hospital, Shanghai Jiaotong University School of Medicine, Shanghai, 200025 China; 2 Department of Infectious Diseases, the Fifth People’s Hospital of Suzhou, Suzhou, 215007 Jiangsu Province China; 3 Discipline of Anatomy and Histology, School of Medical Sciences and The Bosch Institute, University of Sydney, Sydney, NSW 2006 Australia; 4 Department of Infectious Diseases, Ruijin Hospital North, Shanghai Jiao Tong University School of Medicine, Shanghai, 200025 China; 5 Discipline of Pathology, School of Medical Sciences and Bosch Institute, Charles Perkin Centre, University of Sydney, Sydney, NSW 2006 Australia

**Keywords:** hMOF, Chronicity, Hepatitis B, Viral replication, Epigenetic regulation

## Abstract

**Background:**

Chronic hepatitis B (CHB) remains a global health dilemma with high morbidity and mortality. Human males absent on the first (hMOF) (a histone acetyltransferase) is responsible for DNA damage repair, tumorigenesis and cell cycle regulation. Persistence of HBV DNA contributes to cirrhosis and hepatocellular carcinoma (HCC) in CHB patients. Histone acetyltransferase enhances HBV replication, however the precise underlying mechanism of hMOF in HBV replication in CHB patients remains to be explored. This study aims to investigate the correlation between hepatic hMOF and HBV DNA replication in CHB patients, and may provide new insights towards the treatment of CHB patients.

**Methods:**

hMOF in liver biopsy (CHB, n = 33 HBeAg^+^; n = 20 HBeAg^−^, and three healthy controls) was determined, using immunohistochemistry, qPCR and Western blot. The correlation between hMOF and HBsAg, as well as, HBeAg were determined.

**Results:**

A positive correlation between hMOF and HBV DNA in overall CHB patients was observed. A distinct positive correlation between hMOF and HBsAg and/or HBeAg in HBeAg^+^ CHB patients was also detected, however not observed between hMOF and HBsAg in HBeAg^−^ CHB patients. No correlation was observed between hMOF and hepatic inflammation severity and fibrotic stage in CHB patients.

**Conclusions:**

Hepatic hMOF might contribute to host HBV clearance in CHB patients and possible pathogenesis.

**Electronic supplementary material:**

The online version of this article (10.1186/s13578-018-0215-5) contains supplementary material, which is available to authorized users.

## Background

Hepatitis B virus (HBV) infection continues to be a global health problem with high morbidity and mortality, despite decades of extensive research into antiviral drugs and vaccines [1]. Chronicity of HBV infection has been attributed to the unique and stable replication system employed by the virus. The episomal viral genome, the covalently closed circular DNA (cccDNA), forms within infected hepatic nuclei, resulting in increased difficulty for host HBV clearance [2]. Chronic hepatitis B infection (CHB) eventuates in irreversible conditions, including cirrhosis, hepatic decompensation, and hepatocellular carcinoma [3]. The precise pathogenesis of CHB is still a major clinical challenge and remains to be explored.

Males absent on the first (MOF) is a member of MYST family of histone acetyltransferases (HATs). Acetylation of H4K16, a component of the lysine group on the N-terminal tail of histone H4, raises the level of X chromosome transcriptional activity in males at twice the level of females, promoting a dosage compensation effect [4]. Human males absent on the first (hMOF) is involved in the transcriptional regulation of genes, DNA damage repair, tumorigenesis, and cell cycle regulation [5]. Abnormal hMOF expression has been detected in many malignancies, including kidney [6], ovarian [7], lung [8], breast [9] and medulloblast [9].

It is well known that HBV DNA is a high-risk factor in the progression of cirrhosis and hepatocellular carcinoma (HCC) in CHB patients [10]. Epigenetic regulation alters the chromatin structure without variation of gene sequences, including gene transcription, recombination, DNA replication, and damage repair [11]. Epigenetic regulation also participates in post-translational modifications of HBV, such as DNA methylation and histone modifications, specifically acetylation of H4K16 [12]. HBV cccDNA, organized into minichromosomes in the nucleus of the host cells by histone and non-histone proteins [13], is the key transcriptional template for HBV RNA. Histone acetyltransferase has been demonstrated to enhance HBV replication, while histone deacetylase has been identified to suppress HBV replication [12]. Furthermore, our previously published data demonstrates that depletion of hMOF represses HBV replication in vitro, leading to a decrease in HBsAg and HBeAg levels [14]. The directly linkage between hMOF and CHB status remains to be explored.

Our current study aims to investigate the correlation between hMOF and HBV DNA replication in the liver of CHB patients. Understanding the correlation between the expression of hMOF and HBV replication may shed light on pharmaceutical development towards the prevention and treatment of CHB patients.

## Methods

### Human subjects

A total of 53 CHB patients were recruited, including 33 HBeAg^+^, 20 HBeAg^−^ CHB patients, and three healthy controls, from December 2012 to December 2014 in the Department of Infectious Diseases, Ruijin Hospital, Shanghai, China. CHB patients aged 18–65 years, were identified as HBV mono-infected with HBsAg^+^ for at least 6 months and were naïve to antiviral treatment and immunotherapy prior to the initiation of the current study [15]. HBeAg^+^ chronic hepatitis B patents are characterized by the presence of serum HBeAg with high levels of HBV DNA, while chronic hepatitis B patients are characterized by the presence of serum antibodies to HBeAg (anti-HBe), and persistent or fluctuating moderate to high levels of serum HBV DNA (often lower than in HBeAg^+^ patients) [16]. Exclusion criteria were: (1) co-infection with HAV, HCV, HDV or HEV; (2) autoimmune liver diseases; (3) non-alcoholic fatty liver diseases; (4) alcoholic liver diseases; (5) congenital metabolic liver disease; (6) evidence of hepatocellular carcinoma (suspicious foci on hepatic ultrasonic examination or CT, or a rising serum level of α-fetoprotein). Liver biopsy (n = 53) was obtained for histological examination.

### Laboratory assay

Serum alanine aminotransferase (ALT) and aspartate aminotransferase (AST) levels were tested routinely, using an automated chemistry analysis system (Beckman Coulter, Fullerton, CA, USA). Serum HBsAg and HBeAg were determined, using commercial enzyme immunoassay kits (AXSYM System; Abbott, Wiesbaden, Germany). Serum HBV DNA level was quantified, using Applied Biosystems PCR system (Prism 7500; Applied Biosystems, Inc., USA), with a lower limit of quantification at 200 IU/mL.

#### Western blot

Western blot was performed as described previously [17]. Briefly, liver tissues were lysed in RIPA buffer (Beyotime, China), and extracted proteins were quantified using a BCA assay (Beyotime, China). Proteins (10 μg) were transferred to PVDF membranes which were then blocked with 20 mL 5% fat-free milk in 1× TBS at room temperature for 1 h. Membranes were incubated with either rabbit anti-human hMOF, 1:2000, (Abcam, Cambridge, UK); or mouse anti-human GAPDH, 1:2000, (Abcam, Cambridge, UK) at 4 °C overnight, followed by secondary antibody (goat anti-rabbit-HRP or goat anti-mouse–HRP, 1:5000 each; Beyotime, China) for 1 h and visualized, using ImageQuant™ LAS 4000 (Fujifilm, Tokyo, Japan).

#### qRT-PCR

Trizol reagent (Invitrogen, USA) was used to extract RNA from liver tissues, with cDNA synthesized, using a reverse transcriptase kit (TaKaRa). qPCR was subsequently conducted in in three independent assays according to the manufacturer’s instruction, using SYBR Green PCR Master Mix (TaKaRa) in duplicates. Relative hMOF expression levels were quantified after normalization to GAPDH as an internal control. The primers used were listed in Table 1.

**Table 1:** Related primer sequences of qRT-PCR

Gene	Related primer sequences
hMOF-forward primer	GAAGGAGCATGAGGCGATCA
hMOF-reverse primer	TTTCGTAGTTCCCGATGTGGAT
GAPDH-forward primer	ATCACTGCCACCCAGAAGAC
GAPDH-reverse primer	ATGAGGTCCACCACCCTGTT

#### Immunohistochemistry

Liver biopsies from 53 CHB patients and three healthy liver transplant donors were collected for immunohistochemistry. Immunohistochemistry was performed as previously described [18], using rabbit anti-human hMOF primary antibody (Abcam, Cambridge, MA, USA). Every test was coupled with a negative control in which the antibody was substituted by the primary rabbit negative control. Immunohistochemical assay and computer-assisted genuine color image analysis system (ImagePro-plus 7.0) was used to quantify objectively the integrated optional density of hepatic hMOF [18, 19].

#### Liver biopsy

Percutaneous liver biopsy was performed under the guidance of ultrasound. All puncture samples were more than 1 cm in length, and at least six portal tracts were contained for evaluation. Liver histopathology was graded by a pathologist independently in a double-blind fashion. Modified Ishak’s histological activity index (HAI) for necroinflammation and the Ishak fibrosis score for fibrosis were used [20]. Remaining liver biopsy samples were stored at − 80 °C for further use.

#### Statistics

Data are presented as mean ± SD. Statistical analysis was performed using SPSS 17.0 statistical software (SPSS Inc, Chicago, IL, USA) and GraphPad Prism 6 (GraphPad Software, San Diego, CA, USA). For normally distributed data, independent-sample *t* test was used for comparisons between two groups. For abnormally distributed data, non-parametric statistics was performed, and Mann–Whitney U test was used for comparisons between two groups. Chi square tests were performed in comparisons between categorical factors. Spearman rank correlation analyses were performed to analyze the association between measured parameters and ranked data, otherwise Pearson correlation analyses were used. All *p* values mentioned were two-sided. All values *p* < 0.05 were considered to be statistically significant.

## Results

### Clinical characteristics

All 53 CHB patients were classified by HBeAg status. Among 33 HBeAg^+^ CHB patients, the level of serum HBsAg, HBeAg, ALT or AST was 20,157 ± 29,156 IU/mL, 689 ± 623 S/CO, 46.06 ± 21.45 IU/L or 35.38 ± 13.69 IU/L, respectively. Conversely, among the 20 HBeAg^−^ CHB patients the level of serum HBsAg, ALT or AST was 1047 ± 1443 IU/mL, 43.10 ± 27.99 IU/L or 34.70 ± 17.52 IU/L, respectively (Table 2).

**Table 2:** Clinical characteristics of CHB patients

	Total (n = 53)	HBeAg^+^ (n = 33)	HBeAg^−^ (n = 20)
Male, n (%)	34 (64.15%)	21 (63.63%)	13 (65.00%)
Age (years)	37.62 ± 9.38	35.12 ± 8.94	41.75 ± 8.79
ALT (IU/L)	44.94 ± 23.90	46.06 ± 21.45	43.10 ± 27.99
AST (IU/L)	35.06 ± 15.11	35.38 ± 13.69	34.70 ± 17.52
HBV DNA (log_10_IU/mL)	5.63 ± 1.97	6.54 ± 1.74	4.14 ± 1.36
HBsAg (IU/mL)	12,838 ± 24,624	20,157 ± 29,156	1047 ± 1443
HBeAg (S/CO)	/	689 ± 623	/

Data was expressed as mean ± standard deviation

### Correlation between hepatic hMOF and serum HBV DNA, HBsAg and/or HBeAg levels

**Fig. 1:**
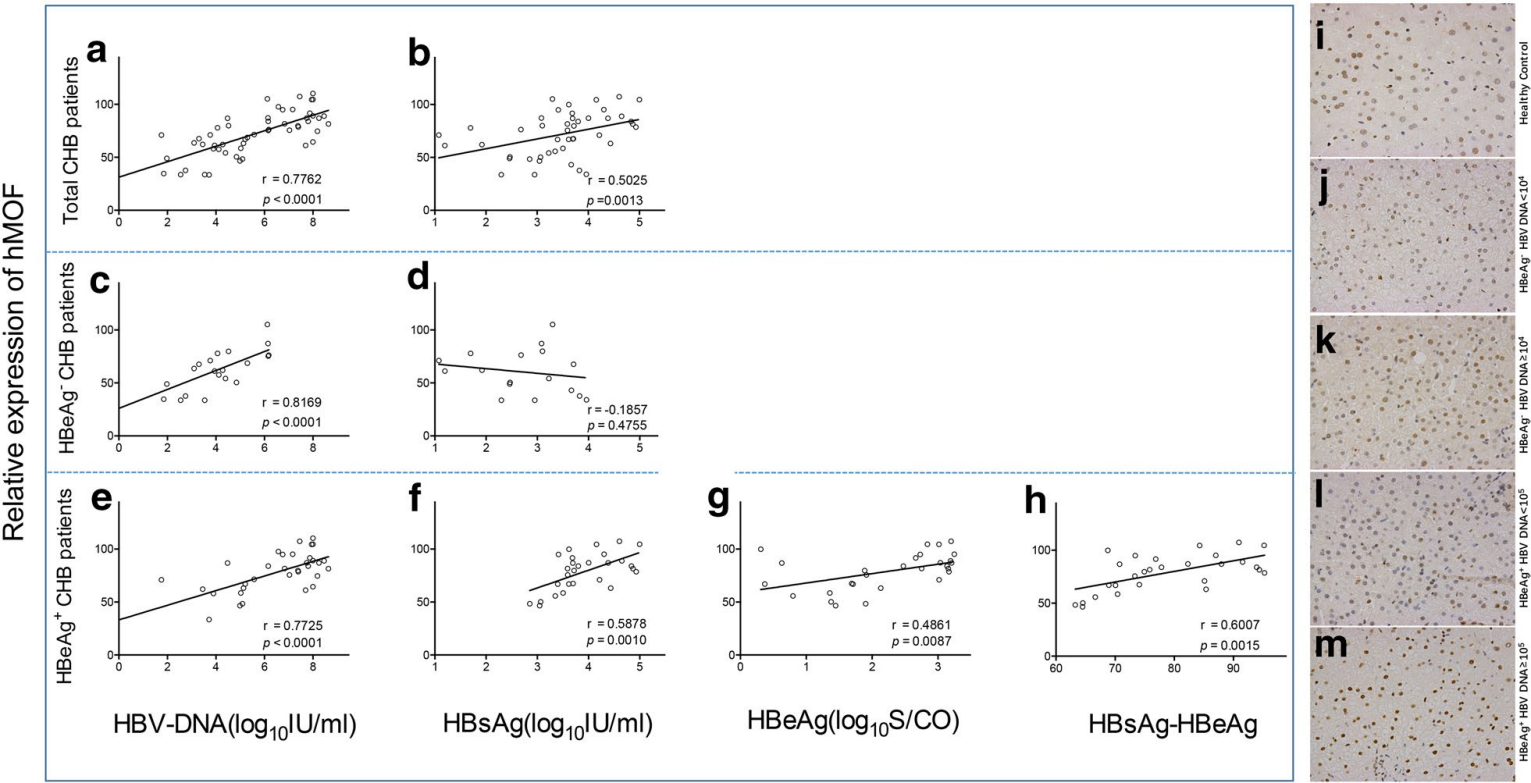
Correlation between hepatic hMOF and serum HBV DNA, HBsAg, and HBeAg levels. Correlation between hepatic hMOF and serum HBV DNA, HBsAg and/or HBeAg levels in CHB patients (**a**, **b**), and HBeAg^−^ CHB patients (**c**, **d**), and HBeAg^+^ CHB patients (**e**–**g**), and correlation between hepatic hMOF and combination of HBsAg–HBeAg in HBeAg^+^ CHB patients (**h**). Representative immunohistochemical micrographs of hepatic hMOF in different liver tissues: Healthy control (**i**); HBeAg^−^ CHB patients with low HBV DNA load (**j**) and high HBV DNA load (**k**); HBeAg^+^ CHB patients with low HBV DNA load (**l**) and high HBV DNA load (**m**)

Among all CHB patients, significant correlation was observed between hepatic hMOF and serum HBV DNA (*p* < 0.001, r = 0.7762, Fig. 1a), or between hepatic hMOF and serum HBsAg (*p* = 0.0013, r = 0.5025, Fig. 1b). Similarly, within 33 HBeAg^+^ CHB patients, a significant correlation was identified between hepatic hMOF and serum HBV DNA (*p* < 0.0001, r = 0.7725, Fig. 1e), hepatic hMOF and serum HBsAg (*p* = 0.0010, r = 0.5878, Fig. 1f), and hepatic hMOF and serum HBeAg (*p* = 0.0087, r = 0.4861, Fig. 1g). Combining HBsAg and HBeAg together, we also found a significant correlation between hepatic hMOF and HBsAg–HBeAg in HBeAg^+^ CHB patients (adjusted *p* = 0.0015, r = 0.6007, Fig. 1h). Furthermore, correlation between hepatic hMOF and serum HBV DNA levels was observed (*p* < 0.0001, r = 0.8169, Fig. 1c) in HBeAg^−^ CHB patients, however no correlation was detected between hepatic hMOF and serum HBsAg levels (*p* = 0.4755, Fig. 1d).

Consistent with the results above, our imunohistochemical analysis demonstrated that hepatic hMOF was markedly upregulated in HBeAg^+^ CHB patients compared with healthy controls (Fig. 1i), both in high HBV DNA load groups (HBV DNA ≥ 1 × 10^5^ IU/mL) (Fig. 1m) and in low HBV DNA load groups (HBV DNA < 1 × 10^5^ IU/mL) (Fig. 1l). Moreover, among HBeAg^+^ CHB patients with high HBV DNA load, hepatic hMOF was 1.4-fold higher than those patients with low HBV DNA load. Similarly, hepatic hMOF was markedly upregulated in high HBV DNA load groups (HBV DNA ≥ 1 × 10^4^ IU/mL) (Fig. 1k) in HBeAg^−^ CHB patients compared with healthy controls. Hepatic hMOF in HBeAg^−^ CHB patients with high HBV DNA load were 1.7-fold higher than those with low HBV DNA load (HBV DNA < 1 × 10^4^ IU/mL) (Fig. 1j). Hepatic hMOF in HBeAg^−^ CHB patients with high HBV DNA load were 2.2-fold higher than healthy controls. However, there was no significant difference of hepatic hMOF between low HBV DNA load of HBeAg^−^ CHB patients and healthy controls.

### Hepatic hMOF in HBeAg^+^ and/or HBeAg^−^ CHB patients

**Fig. 2:**
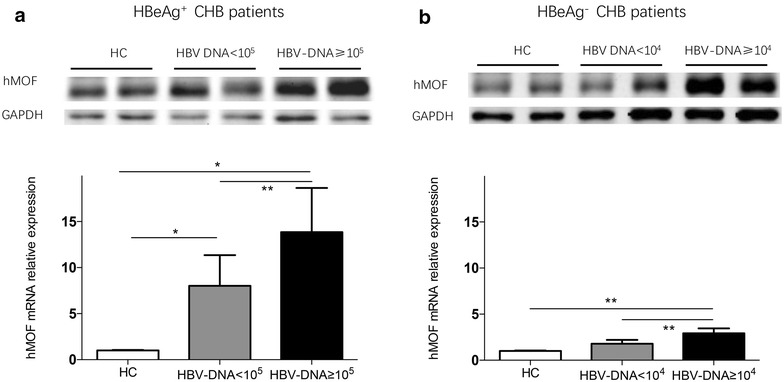
Hepatic hMOF in HBeAg^+^ and/or HBeAg^−^ CHB patients and healthy controls. Hepatic hMOF production was presented with Western blot and quantitative analysis of hepatic hMOF mRNA expression was measured by qPCR, from HBeAg^+^ CHB patients (**a**) and HBeAg^−^ CHB with high HBV DNA load (HBV DNA ≥ 10^5^ IU/mL, 10 cases) and with low HBV DNA load (HBV DNA < 10^5^ IU/mL, 10 cases), and healthy controls (HC) (three cases), and HBeAg^−^ CHB (**b**) with high HBV DNA load (HBV DNA ≥ 10^4^ IU/mL, 10 cases) and with low HBV DNA load (HBV DNA < 10^4^ IU/mL, 10 cases), and healthy controls (HC) (three cases). The significant difference is expressed as *p < 0.05, **p < 0.01

Western blot analysis and qRT-PCR were conducted to illustrate hepatic hMOF expression both in HBeAg^+^ CHB patients (Fig. 2a) and HBeAg^−^ CHB patients (Fig. 2b). Hepatic hMOF expression was observed to be significantly higher with both high HBV DNA load (HBV DNA ≥ 1 × 10^5^ IU/mL, 10 cases) (2.4-fold) and low HBV DNA load (HBV DNA < 1 × 10^5^ IU/mL, 10 cases) (1.7-fold) in HBeAg^+^ CHB patients compared to healthy controls (three cases). Furthermore, hepatic hMOF was 1.7-fold higher with high HBV DNA load compared with those with low HBV DNA load (*p* < 0.01) in HBeAg^+^ CHB patients. Similarly, hepatic hMOF expression was markedly higher in HBeAg^−^ CHB patients with high HBV DNA load (HBV DNA ≥ 1 × 10^4^ IU/mL, 10 cases) than these with low HBV DNA load (HBV DNA < 1 × 10^4^ IU/mL, 10 cases) and healthy controls (three cases) (2.0-fold and 1.2-fold, respectively). There is no significant difference of the hepatic hMOF expression between HBeAg^−^ CHB patients with low HBV DNA load and healthy controls.

### Correlation between hepatic hMOF and severity of inflammation, or stage of fibrosis of liver

**Fig. 3:**
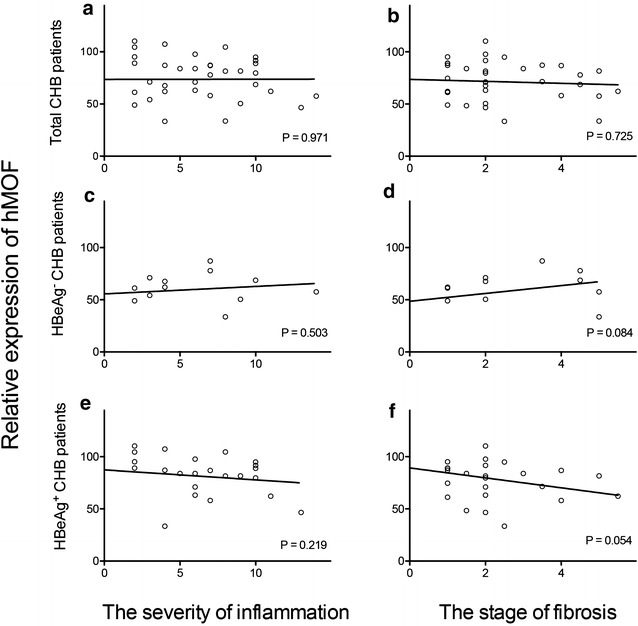
Correlation between hepatic hMOF and severity of inflammation as well as, stage of fibrosis of liver. Correlations between hepatic hMOF and severity of inflammation and stage of fibrosis of liver was described in CHB patients (**a**, **b**), in HBeAg^−^ CHB patients (**c**, **d**) and in HBeAg^+^ CHB patients (**d**, **e**)

Among CHB patients, no significant correlation was observed between hepatic hMOF and severity of inflammation or hepatic hMOF and stage of fibrosis of liver (Fig. 3a or b). Furthermore, no significant correlation was observed between hepatic hMOF and severity of inflammation or hepatic hMOF and stage of fibrosis of liver in HBeAg^+^ CHB patients (Fig. 3e, f). Additionally, no significant correlation was observed between hepatic hMOF and severity of inflammation or between hepatic hMOF and severity and stage of fibrosis of liver in HBeAg^−^ CHB patients (Fig. 3c, d).

### Correlation between serum HBV DNA, HBsAg and/or HBeAg levels and histological damage of liver

In total CHB patients, no significant correlation was observed between serum HBV DNA, HBsAg and/or HBeAg levels and hepatic inflammation severity, or fibrotic stage of liver (Additional file 1: Figure S1A–D). Similarly, there is no significant correlation between serum HBV DNA, HBsAg and/or HBeAg status and hepatic inflammation severity or fibrotic stage of liver neither in HBeAg^+^ CHB patients (Additional file 1: Figure S1E–H), nor in HBeAg^−^ CHB patients (Additional file 1: Fig S1I–L).

## Discussion

HBV infection remains a major clinical challenge worldwide with continued economic burden, as current anti-viral therapy is not completely effective [3]. The precise underlying mechanism of persistent HBV replication in CHB patients, and the associated significant risk of developing liver cirrhosis and HCC, requires further investigation. It is therefore necessary to explore the possible mechanisms involved in the persistence of HBV in CHB patients, and identify potential novel target(s) for the development of pharmaceutical treatments of such a devastating disease.

In our present study, we detected the correlation between hepatic hMOF and HBV DNA replication in CHB patients, suggesting that hMOF plays an important role in regulating HBV replication. This is consistent with our previous study that demonstrated HBV replication is markedly repressed in vitro [14]. However, the role of hMOF in HBV replication is still unclear. HBV cccDNA is a key event in the process of HBV replication within the infected hepatocytes post-translational modifications (PTMs) via acetylation [12, 13, 21]. There is no direct linkage between upregulation of hMOF and cccDNA in CHB patients.

One of the key findings of our current study was the significant correlation between hepatic hMOF and serum HBV DNA in total CHB patients. hMOF serves as an enzyme in histone acetylation in the process of post-translational modifications. Serum HBV DNA appears to be a useful marker of HBV replication [22], with cccDNA and relaxed circular (RC) DNA consisting of total intrahepatic HBV DNA [23]. hMOF also participates in cccDNA mediated post-translational modifications (acetylation) [21]. The data from our current study demonstrated that a correlation between hepatic hMOF and circulating HBV DNA in CHB patients in vivo. Our data is supported by our previous study in vitro that depletion of hMOF obviously represses HBV replication, leading to a decrease in HBsAg and HBeAg levels [14]. Our data further suggests that hMOF plays a critical role in enhancing HBV replication, including promoting HBV replication in hepatocytes. The precise underlying mechanism of how hMOF specifically regulates HBV replication remains to be explored.

In our present study, we observed close correlations between hepatic hMOF and serum HBsAg, and HBeAg levels in total CHB patients and in HBeAg^+^ CHB patients, however not in HBeAg^−^ CHB patients. hMOF may be used as a potential biomarker in predicting HBV replication, particularly in HBeAg^+^ patients who usually express high loads of HBV due to their low immune status [16]. It is well documented that levels of circulating HBsAg and HBeAg reflect the activity of HBV replication, although not intrahepatic HBV DNA level [24]. Thus, our current data is in line with HBV DNA, suggesting that hepatic hMOF reflects viral replication, particularly in HBeAg^+^ CHB patients.

The discrepant correlation between serum HBsAg and HBV DNA/cccDNA has been described in previous studies [25–27]. Serum HBsAg levels correlate with cccDNA [25] in HBeAg^+^ CHB patients [26] but not in HBeAg^−^ CHB patients [27], suggesting that HBsAg may not be a comprehensive predictor of HBV replication in HBeAg^−^ CHB patients. The upregulation of serum HBsAg expression involves the integrated viral genome in addition to the amount of cccDNA in infected hepatocytes [28]. Serum HBsAg levels can therefore reflect cccDNA concentration in HBeAg^+^ CHB patients during different antiviral therapy phases [25, 29], suggesting that serum HBsAg may be used as a cccDNA predictor when cccDNA levels are high. In HBeAg^−^ CHB patients, the production of cccDNA is reduced by the immune clearance [23], thus the contribution of cccDNA to HBsAg production may be lower than that of HBeAg^+^ CHB patients. Such reports are in line with our current findings, where no significant correlation was observed between hepatic hMOF and serum HBsAg level in HBeAg^−^ CHB patients.

We hypothesize that hMOF upregulates HBV replication more effectively in HBeAg^+^ CHB patients than that in HBeAg^−^ CHB patients. We don’t have firm evidence that hMOF is the key molecule determining host immunity against HBV clearance. Nevertheless, our data invites speculation that the interaction between hMOF and the immune system determines host immunity against HBV clearance in HBeAg^+^ patients, who have relatively low host defense. The precise underlying mechanism is being determined.

To determine if hMOF contributes to hepatic damage during the development of CHB, correlation between hMOF and hepatic inflammation/fibrosis was investigated. Interestingly, there was no significant correlation between hMOF and severity of inflammation, nor stage of fibrosis of the liver in CHB patients (with/without HBeAg^+^). These data suggest hMOF may not be an ideal biomarker for hepatic inflammation or fibrosis. A possible explanation is that hepatic damage is considered to be caused by host cellular immunity against HBV infected hepatocytes, rather than the direct HBV cytopathic effect [30], or that hMOF might not be sensitive at the chronic stage. A close correlation between HBV DNA load and intrahepatic hMOF perhaps can explain that there was not a direct correlation between hMOF and intrahepatic histology, nor serological viral markers, such as HBsAg and HBeAg. Our explanation is consistent with others, showing no significant correlation between serum HBV DNA levels and liver histology in terms of necroinflammation and fibrosis in HBeAg^+^ CHB patients [31, 32]. Consistently, there was no significant correlations between serum HBV DNA level, HBsAg and/or HBeAg and severity of inflammation, or stage of fibrosis of liver neither in total CHB patients, nor in HBeAg^+^ CHB patients or in HBeAg^−^ CHB patients.

## Conclusion

Hepatic hMOF may be responsible for the persistence of chronicity of HBV infection by promoting HBV replication in CHB patients, particularly in HBeAg^+^ patients. Such information may provide new insights into pharmaceutical development in prevention and treatment of CHB. Future studies will explore the underlying mechanism hMOF regulates HBV replication in larger cohort.

## Additional file

**Additional file 1.** Correlation between serum HBV DNA level, and HBsAg status and severity of inflammation and stage of fibrosis of liver.

## Abbreviations

CHB: chronic hepatitis B subjects without cirrhosis and liver failure
HBV: hepatitis B virus
hMOF: human males absent on the first
HCC: hepatocellular carcinoma
cccDNA: covalently closed circular DNA
ALT: alanine aminotransferase
AST: aspartate aminotransferase
HATs: histone acetyltransferases
PTMs: post-translational modifications
RC DNA: relaxed circular DNA
