# Supplementary material for: Correlation between hepatic human males absent on the first (hMOF) and viral persistence in chronic hepatitis B patients
Source: Cell Biosci. 2018 Feb 20;8:14. doi: 10.1186/s13578-018-0215-5 (PMC5819663; doi:10.1186/s13578-018-0215-5)

**Additional file 1 for**

**Correlation between hepatic human males absent on the first (hMOF) and viral persistence in chronic hepatitis B patients**

**Figure S1. Correlation between serum HBV DNA level, and HBsAg status and severity of inflammation and stage of fibrosis of liver.** Correlations between severity of inflammation and stage of fibrosis of liver and serum HBV DNA and HBsAg were observed in CHB patients (A-D), in HBeAg^-^ CHB patients (E-H), and in HBeAg^+^ CHB patients (I-L).


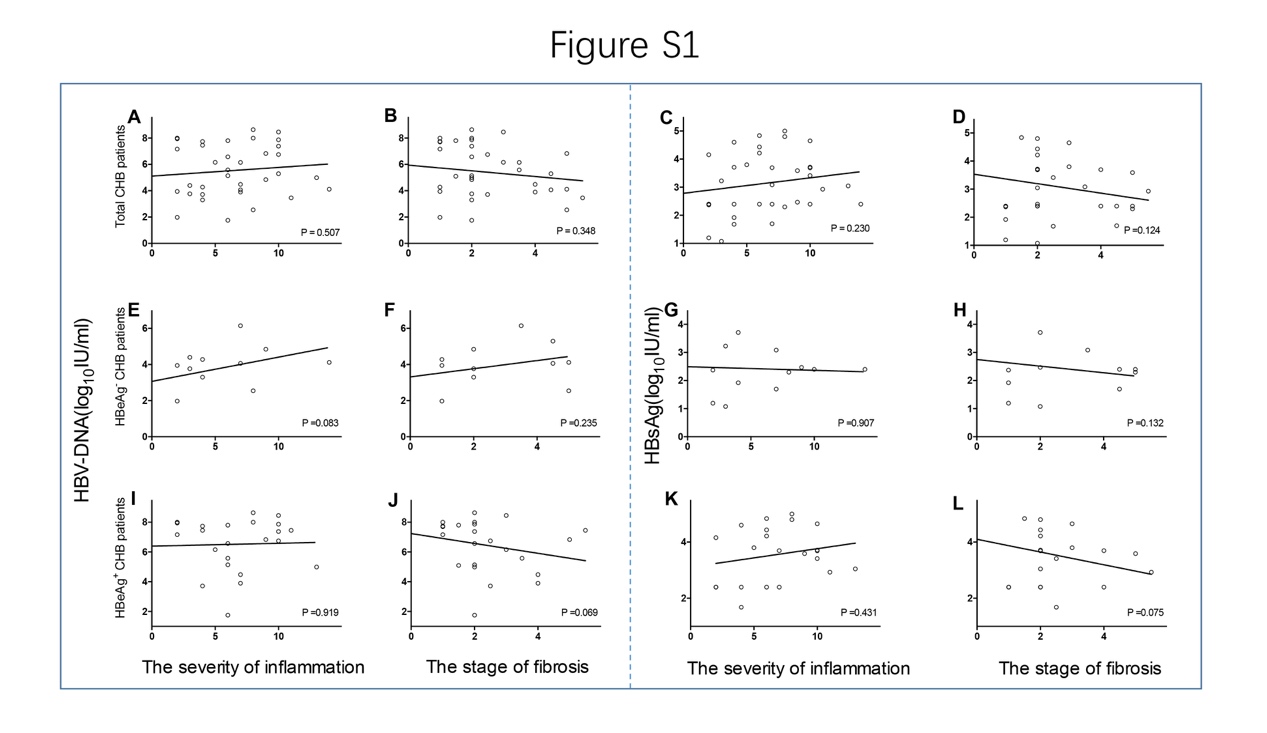

Supplement: Supplementary file 1 — Additional file 1. Correlation between serum HBV DNA level, and HBsAg status and severity of inflammation and stage of fibrosis of liver. [file 13578_2018_215_MOESM1_ESM.docx]
